# Relationship of Problematic Smartphone Use, Sleep Quality, and Daytime Fatigue Among Quarantined Medical Students During the COVID-19 Pandemic

**DOI:** 10.3389/fpsyt.2021.755059

**Published:** 2021-11-10

**Authors:** Chi Zhang, Ping Zeng, Joshua Tan, Siwei Sun, Minghao Zhao, Ju Cui, Guifang Zhang, Jinzhong Jia, Deping Liu

**Affiliations:** ^1^The Key Laboratory of Geriatrics, Beijing Institute of Geriatrics, Institute of Geriatric Medicine, Chinese Academy of Medical Sciences, Beijing Hospital, National Center of Gerontology of National Health Commission, Beijing, China; ^2^School of Basic Medicine, Peking University Health Science Center, Beijing, China; ^3^Peking University Sixth Hospital, Peking University Institute of Mental Health, NHC Key Laboratory of Mental Health, National Clinical Research Center for Mental Disorders, Beijing, China; ^4^Graduate School Health Science Center of Peking University, Secretariat Office of National Steering Committee for Medical Professional Degree Education, Beijing, China; ^5^Department of Cardiology, Beijing Hospital, National Center of Gerontology, Institute of Geriatrics Medicine, Chinese Academy of Medical Sciences, Beijing, China

**Keywords:** problematic smartphone use, sleep quality, fatigue, mediating effect, COVID-19

## Abstract

**Background:** The COVID-19 pandemic brought about great transformation to medical education mode. Although mobile communication devices played a crucial role in online learning among quarantined university students, the potential smartphone addition problems, negative health behaviors, and psychological symptoms need considerable attention. This study examined the relationship of problematic smartphone use (PSU), sleep quality, and daytime fatigue among medical students.

**Methods:** A web-based survey was conducted in six polyclinic hospitals in Beijing between February and May 2020. 1016 participants (26.01 ± 2.46 years, 65.16% female) completed self-report measurements including Short Version Smartphone Addiction Scale (SAS-SV), Athens Insomnia Scale (AIS), and Subjective Fatigue Scale (FS). Spearman correlation coefficients and multiple regression models were used to analyze the association among PSU, sleep quality, and daytime fatigue. We used structural equation modeling to test the mediating effect of sleep quality between PSU and daytime fatigue.

**Results:** 49.70% of the participants had PSU. Significant positive correlations were found among SAS-SV, AIS, and FS scores (*r* = 0.35–0.61, *P*_*S*_ < 0.001). Subjects with PSU were more likely to report sleep disturbance (β = 1.07, *P* < 0.001, OR = 2.91, 95%CI = 2.17–3.91), physical fatigue (β = 1.16, *P* < 0.001, OR = 3.18, 95%CI = 2.45–4.15), and mental fatigue (β = 0.88, *P* < 0.001, OR = 2.42, 95%CI = 1.86–3.14). The indirect effect of PSU on physical fatigue and mental fatigue mediated by sleep quality accounted for 50.03 and 45.43% of the total effect, respectively.

**Conclusions:** PSU was significantly associated with sleep disturbance and fatigue among medical students during the COVID-19 pandemic. Sleep quality mediated the relationship between PSU and daytime fatigue. Our results provide valuable information for maintaining medical students' health status and constructing online education structures.

## Introduction

Since December 2019, an acute respiratory infectious disease caused by a novel coronavirus (SARS-CoV-2) broke out in Wuhan, China. The World Health Organization named it Coronavirus Disease 2019 (COVID-19) and declared on March 11 that the COVID-19 outbreak was a global pandemic (1). Recently emerged SARS-CoV-2 delta variants spreading in China and worldwide gave rise to a new wave of pandemics (2). The COVID-19 pandemic, a public health emergency, has had significant impacts on China's healthcare and medical education system (3), as governments adopted strict control measurements to require people to stay at home for social distancing (4). Many scholars have expressed their concerns about the online medical education in current and future pandemics (5, 6). One critical issue is that with the rapid development of communication technology, smartphone addiction and related clinical symptoms may surge among student populations. A cross-sectional online survey conducted by Chen and colleagues showed that primary school children who had psychological distress during the COVID-19 outbreak might spend longer time on Internet-related activities (7). The relationship between PSU and psychological distress has been influenced by the COVID-19, which has been confirmed by Chen's another longitudinal Study (8). Some scholars identified the overuse of smartphones as a hidden crisis during the pandemic (9), and this issue has been highlighted by recent literatures in China. A recent national survey among 746, 217 Chinese college students showed that the risks of developing depression and anxiety disorders increased with the exposure time to electronic devices (10). Another cross-sectional study showed that the level of COVID-19 related anxiety symptoms was correlated with the severity of problematic smartphone use (PSU) among Chinese adults (11). Many studies indicated that smartphone overuse directly impacts daytime functions and sleep quality (12–14), while the relationship has not been identified under the influence of the COVID-19 pandemic among medical students.

During the pandemic, medical students under quarantine relied heavily on the Internet and smartphone technology to complete heavy academic tasks and obtain the COVID-19 related information. Since medical students are more sensitive when tracking the pandemic information using smart deceives, their psychological patterns may differ from those of the general population (15). Thus, more studies about medical students are needed to cope with the transformation of the health care and education system. Colleges in Beijing have implemented measures to delay college students' return to school and required them to stay at home or in dormitories since January 2020. During an extended period of home isolation, university students' various living habits might change, among which the overuse of smartphones along with the resulting physical and mental health problems needs to be considered. Problematic smartphone use has been previously defined as excessive use of a smartphone that is accompanied by functional impairments in daily living, and substance addiction-like symptoms (16). PSU can cause many detrimental physical and psychological disorders and it has become a mental health threat to university students (17). A longitudinal study showed that the initial level of problematic use of smartphone/internet increased the psychological distress among university students in Hong Kong (18). Fatigue, defined as a subjective feeling of tiredness, weakness and discomfort, is now widely recognized as a premorbid state and causative factor (19). In clinical practice, physical fatigue and mental fatigue have specific symptoms.

Recent studies revealed that the intensity and time of electronic devices usage were relatively high during the COVID-19 pandemic, along with the increased incidence of smartphone addiction (20, 21), which could induce adverse behavioral and health outcomes (22, 23). Several studies have discussed the psychological and clinical mechanisms of the smartphone addiction issues, which are beneficial to support our hypotheses. In Billieux's pathway models, psychological, biological, social, and environmental factors play multiple roles in predicting PSU and are associated with various dysfunction symptoms (24). According to the Person-Affect-Cognition-Execution Model (I-PACE) of Internet addiction, individual factors such as physiology, personality, emotion, cognition, and executive function can significantly predict Internet addiction (25). In a study on undergraduate students, a combination of alexithymia, dissociative experiences, low self-esteem, and impulse dysregulation was confirmed to be a potential risk factor for Internet Addiction (26). As the Compensatory Internet Use Theory (CIUT) described, if individuals are in a negative situation, they may escape reality by surfing the internet, thus increasing the chance of addiction symptoms (27). The COVID-19 pandemic can be regarded as an unanticipated hostile incident, and people's mental health has been adversely affected, which further aggravates the possibility of dependence on smartphones (28). In addition to the above psychological models, clinical mechanisms are beneficial to support our theoretical model regarding the relationship of PSU, sleep quality, and daytime fatigue. Blue light and electromagnetic radiation emitted by smartphones directly cause harm to users' eyesight, neck, and spine (14, 29). Suppression of melatonin secretion caused by light stimulation at night is also a commonly recognized physiological pathway that results in sleep deprivation (30). As the skeletal muscle is a peripheral clock organ closely related to circadian rhythm, sleep disturbance may cause daytime physical symptoms by inhibiting mitochondrial activity (31). Besides, abnormal cortisol and hypothalamic-pituitary-adrenal cortex (HPA) axis functions have been confirmed as neuroendocrinology pathways of the association between sleep quality and mental fatigue (32). Based on the above mechanisms, we hypothesize that sleep disturbance is not only a direct adverse outcome of PSU, but also an important mediator of the relationship between PSU and daytime fatigue. Previous studies also showed that sleep quality played an intermediary role between smartphone overuse and various health issues (33–35). Thus, we built a theoretical partial mediation model and the following four hypotheses will be tested in the current study.

*Hypothesis 01 (H01): PSU level is positively correlated with sleep disturbance*.
*Hypothesis 02 (H02): PSU level is positively correlated with daytime fatigue*.
*Hypothesis 03 (H03): Sleep quality is negatively correlated with daytime fatigue*.
*Hypothesis 04 (H04): Sleep quality mediate the relationship between PSU and daytime fatigue*.

## Materials and Methods

### Participants and Ethics

Participants were 1,016 full-time medical postgraduates from six polyclinic hospitals affiliated to Peking University Health Science Center or Peking Union Medical College in Beijing. Data was collected from the Beijing Hospital (*n* = 208), the First Hospital of Peking University (*n* = 112), the People's Hospital of Peking University (*n* = 148), the Third Hospital of Peking University (*n* = 189), the Peking Union Medical College Hospital (*n* = 251), and the Cancer Hospital of the Chinese Academy of Medical Sciences (108). In each hospital, more than 50% of the total graduate students were recruited. The Ethics Committee of Beijing Hospital approved the study protocol (2020BJYEC-231-01).

### Procedures

Since college students in Beijing were quarantined at home or at school until July 2020, a cross-sectional online questionnaire survey was conducted between February and May 2020 through a widely used social network application, “WeChat.” Data was collected by hospital administrators. The background and purpose of the survey, as well as the data consent were explained on the first page of the questionnaire. Each respondent could receive the feedback report *via* email and get compensation ranging from 3 to 10 *yuan* after submission. 1108 questionnaires were collected, and questionnaires with one of the following conditions were considered invalid: the response time was less than 5 min (*n* = 28); the individual's demographic information could not be identified (*n* = 8); all questions in the three scales used in this study were all answered repeatedly (*n* = 56). A total of 92 invalid questionnaires were excluded, and 1016 valid questionnaires were included in the final analysis, with an effective recovery rate of 91.70%. The education level was divided into master's and doctoral; the degree type was divided into academic and professional. An annual household income of <100,000 yuan was considered poor. The specialties were clinical medicine and others (including biology, preventive medicine, nursing, etc.). The student's relationship with the tutors was collected using a positive likert-5 single question (1–3 points = “bad”; 4–5 points = “good”). If the subjects self-reported regular exercise, they were defined as having exercise habits; and those who self-reported occasional exercise or no exercise were defined as having no exercise habit.

### Measurements

#### Short Version Smartphone Addiction Scale

The 10-item Short Version Smartphone Addiction Scale (SAS-SV) developed by Kwon et al. was used to measure PSU (36). The scale consists of ten positive 6-point Likert questions describing the usage of smartphones, and the summed score ranges from 10 to 60. An example question was “Having my smartphone in my mind even when I am not using it.” The subjects were asked how much they agreed with each question (1 point = “disagree”; 6 points = “agree”). According to the threshold recommended for student populations, males with a summed score ≥ 31 and females with a summed score ≥ 33 were identified with PSU. The SAS-SV is the most widely used instrument to assess PSU and has been proved to have good reliability and validity in the Chinese population (37). In this study, the Cronbach's α coefficient of the scale is 0.91.

#### Athens Insomnia Scale

Sleep quality was measured with the Athens Insomnia Scale (AIS), a self-assessment psychometric instrument including eight 6-point Likert items with a total score of 0–24. Eight items in the AIS are related to sleep induction, awakenings during the night, final awakening, total sleep duration, sleep quality, well-being, functioning capacity, and sleepiness during the daytime. The Chinese version of AIS has been confirmed to be reliable and valid among adolescents and adults (38). As previous studies suggested, individuals with a summed AIS score ≥ 6 were classified as having sleep disturbance (39). In this study, the Cronbach's α coefficient of AIS was 0.87.

#### Subjective Fatigue Scale

Daytime fatigue was measured with a 14-item subjective fatigue scale (FS) developed by Chalder et al. (40). The FS consists of 14 two-category fatigue symptom self-assessment items and the scale showed appropriate validity for Chinese populations (41). The answer “yes” is counted as 1 point, the answer “no” is counted as 0, and the total score ranges from 0 to 14. Higher summed FS scores indicate a higher risk of daytime fatigue. Considering the lack of consensus on the cutoff value of the FS, we regard a summed score higher than or equal to 7 as a threshold of daytime fatigue. In the FS, 8 items reflected physical fatigue, and subscale score ≥4 was used as a cutoff; the last 6 items reflected mental fatigue, and subscale score ≥3 was used as a cutoff. In this study, the Cronbach's α coefficient of the FS was 0.84.

### Statistical Analysis

Continuous variables were described as the mean ± standard deviation (M ± SD), and categorical variables were described as numbers with percentage [n (%)]. Student's *t*-test and χ^2^ test were used to compare the differences of SAS-SV scores across subgroups. Spearman correlation coefficient was used to analyze the association among SAS-SV, AIS, and FS scores. Multiple logistic regressions were used to analyze the association between PSU and the risk of fatigue and sleep disturbance. Subjects without PSU were used as the reference group and potential confounders were adjusted. We conducted a structural equation model (SEM) with robust weighted least squares estimation to test the mediating effect of sleep quality between PSU and daytime fatigue. A bias-corrected bootstrap method was used to test the significance of effect values in the SEM, and 1,000 random samples were put back from the original sample to calculate the 95% confidence intervals. Effect values were statistically significant if the 95% confidence interval did not include 0. Statistical analyses were performed using SPSS 24.0 version and Mplus 8.0 version. Statistical significance was accepted at the two-sided 0.05 level.

## Results

### Demographic Characteristics

A total of 1016 graduate students completed the questionnaire with an average age of 26.01 ± 2.46 years. Among the participants, 65.16% were female, 61.81% were master's students, 55.02% had a professional degree type, 77.46% majored in clinical medicine, 31.69% were first-year graduate students, and 34.55% lived in rural areas.

### Problematic Smartphone use Status

The mean SAS-SV score of the participants was 32.73 ± 9.85, and 49.70% had PSU. As Table 1 showed, participants who had PSU were more likely to be male, have professional degree type, non-clinical medicine major, lower household income, live in rural areas, have a bad relationship with tutors, and have no exercise habits (*Ps* < 0.05).

**Table 1:** Characteristics and PSU status of 1,016 medical students.

**Characteristics**		***N***	**PSU** **(*n* = 505)**	**Non-PSU** **(*n* = 511)**	***t*/χ^2^**	***P*-value**
Age (mean ± SD)		1016	25.75 ± 2.35	26.25 ± 2.54	3.244	0.001
Gender [*n* (%)]	Male	354	191(53.95)	163(46.05)	3.929	0.048
	Female	662	314(47.43)	348(52.57)		
Education [*n* (%)]	Master	628	316(50.32)	312(49.68)	0.248	0.618
	Doctoral	388	189(48.71)	199(51.29)		
Degree [*n* (%)]	Academic	457	201(43.98)	256(56.02)	10.901	0.001
	Professional	559	304(54.38)	255(45.62)		
Residence [*n* (%)]	Rural	351	194(55.27)	157(44.73)	6.654	0.009
	Urban	665	311(46.77)	354(53.23)		
Household Income [*n* (%)]	Poverty	523	287(54.88)	236(45.12)	11.551	<0.001
	Non-poverty	493	218(44.22)	275(55.78)		
Major [*n* (%)]	Clinical medicine	787	370(47.01)	417(52.99)	10.153	0.001
	Others	229	135(58.95)	94(41.05)		
Relationship with tutors [*n* (%)]	Good	860	415(48.26)	445(51.74)	4.718	0.029
	Bad	156	90(57.69)	66(42.31)		
Exercise habits [*n* (%)]	Yes	392	157(40.05)	235(59.95)	6.315	0.007
	No	624	393(62.98)	231(37.02)		
Sleep disturbance [*n* (%)]	Yes	717	411(57.32)	306(42.68)	57.607	<0.001
	No	299	94(31.44)	205(68.56)		
Daytime fatigue [*n* (%)]	Yes	407	268(65.85)	139(34.15)	71.741	<0.001
	No	609	237(38.92)	372(61.08)		
Physical fatigue [*n* (%)]	Yes	568	354(62.32)	214(37.68)	83.291	<0.001
	No	448	151(33.71)	297(66.29)		
Mental fatigue [*n* (%)]	Yes	443	278(62.75)	165(37.25)	54.004	<0.001
	No	573	227(39.62)	346(60.38)		

*PSU, problematic smartphone use; SD, standard deviation*.

### Associations Among SAS-SV, AIS, and FS Scores

The mean score of AIS was 8.09 ± 4.59, and 70.57% of the subjects had sleep disturbance. The mean score of FS was 6.42 ± 3.74, and 40.06% had daytime fatigue. Specifically, 55.91% of the subjects had physical fatigue, and 43.60% had mental fatigue, respectively. As Table 2 showed, the SAS-SV score was positively correlated with the AIS score (*r* = 0.38, *P* < 0.001) and fatigue scores (*r* = 0.35–0.41, *P* < 0.001). The AIS scores were positively correlated with fatigue scores (*r* = 0.48–0.61, *P* < 0.001).

**Table 2:** Correlation coefficients among SAS-SV, AIS, and FS scores.

**Measurement**	**α**	**Mean**	**SD**	**1**	**2**	**3**	**4**	**5**
1. Problematic smartphone use	0.91	32.73	9.85	1.00				
2. Sleep disturbance	0.87	8.09	4.59	0.38^***^ (0.34^***^)	1.00			
3. Daytime fatigue	0.84	6.42	3.74	0.41^***^ (0.39^***^)	0.61^***^ (0.55^***^)	1.00		
4. Physical fatigue	0.81	4.16	2.45	0.37^***^ (0.35^***^)	0.58^***^ (0.54^***^)	0.92^***^ (0.90^***^)	1.00	
5. Mental fatigue	0.72	2.26	1.77	0.35^***^ (0.39^***^)	0.48^***^ (0.51^***^)	0.84^***^ (0.81^***^)	0.56^***^ (0.52^***^)	1.00

*** *P < 0.001*.

*α: Cronbach's alpha coefficient*.

*Partial correlation coefficients are shown in parentheses, controlling for age, gender, degree type, household income, major, residence, relationship with tutors, and exercise habits*.

*SAS-SV, Short Version Smartphone Addiction Scale; AIS, Athens Insomnia Scale; FS, Subjective Fatigue Scale; SD, standard deviation*.

In the multiple logistic regression analyses (Table 3), SAS-SV, AIS, and FS scores were involved as dichotomous variables according to corresponding cutoffs. Gender, degree type, household income, major, residence, relationship with tutors, and exercise habits were involved as co-variables. After adjusting for potential confounders, subjects with PSU were more likely to report sleep disturbance (β = 1.07, *P* < 0.001, OR = 2.91, 95%CI = 2.17–3.91), daytime fatigue (β = 1.10, *P* < 0.001, OR = 2.99, 95%CI = 2.29–3.90), physical fatigue (β = 1.16, *P* < 0.001, OR = 3.18, 95%CI = 2.45–4.15), and mental fatigue (β = 0.88, *P* < 0.001, OR = 2.42, 95%CI = 1.86–3.14) than those without PSU.

**Table 3:** Logistic regression analyses of PSU on sleep disturbance and fatigue.

**Models**		**Crude model**				**Adjusted model^Δ^**			
		**β**	**SE**	**OR**	**95%CI**	**β**	**SE**	**OR**	**95%CI**
Sleep disturbance	PSU	1.47	0.11	2.92	2.21–3.89	1.07	0.15	2.91	2.17–3.91
	Non-PSU	Ref.		1		Ref.		1	
Daytime fatigue	PSU	0.12	0.09	3.02	2.32–3.93	1.1	0.14	2.99	2.29–3.90
	Non-PSU	Ref.		1		Ref.		1	
Physical fatigue	PSU	0.85	0.09	3.25	2.51–4.21	1.16	0.13	3.18	2.45–4.15
	Non-PSU	Ref.		1		Ref.		1	
Mental fatigue	PSU	0.21	0.08	2.56	1.98–3.31	0.88	0.12	2.42	1.86–3.14
	Non-PSU	Ref.		1		Ref.		1	

Δ *Adjusted model was adjusted for age, gender, degree type, household income, major, residence, relationship with tutors, and exercise habits*.

*β: Standardized regression coefficient*.

*Ref: Participants with non-PSU were reference category*.

*PSU, problematic smartphone use; SE, standard error; OR, odds ratio; CI: confidence interval*.

### Mediation Effect of Sleep Disturbance Between PSU and Fatigue

**Figure 1:**
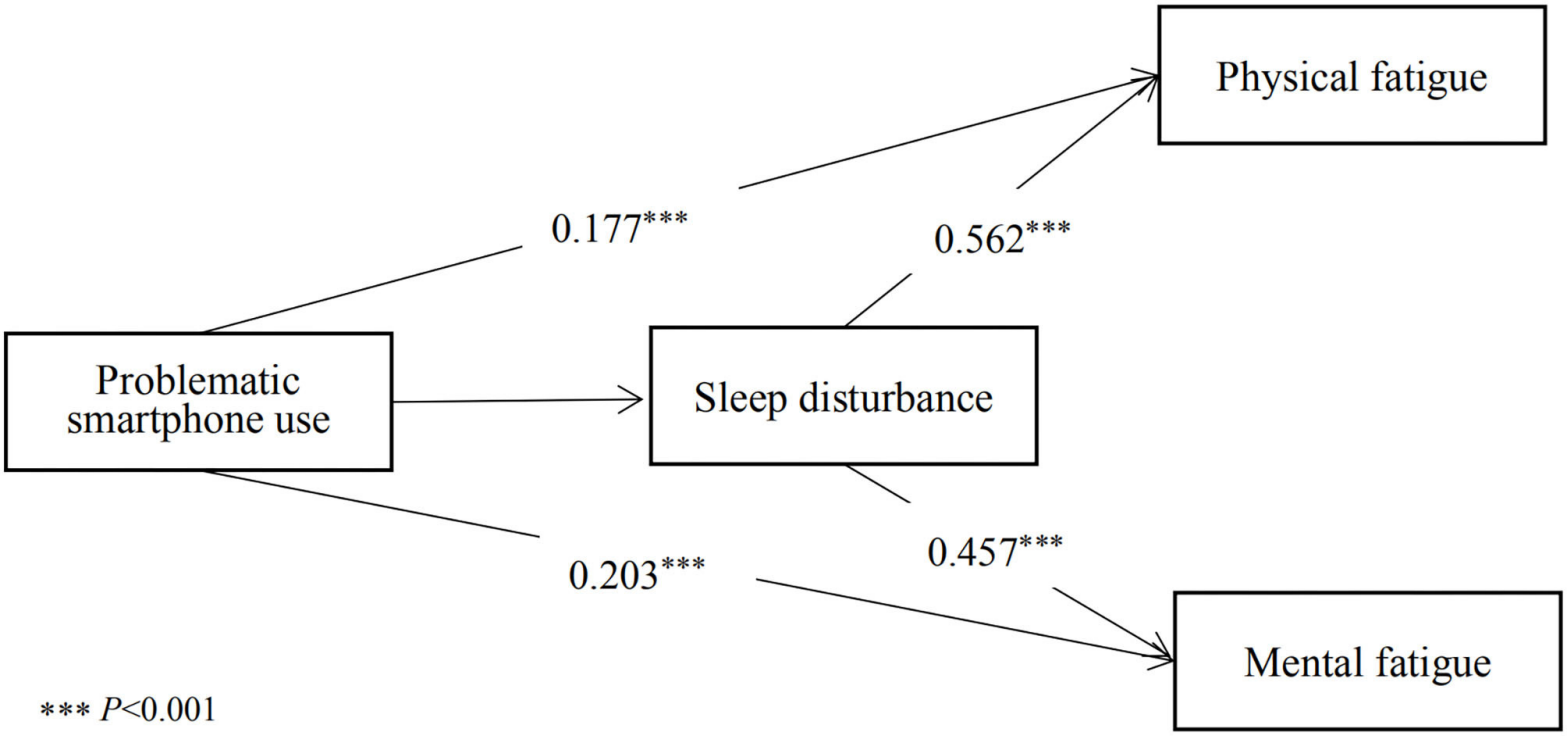
Mediation models of problematic smartphone use, sleep disturbance, physical fatigue, and mental fatigue.

According to the theoretical hypothesis, a partial mediation model of sleep disturbance between PSU and daytime fatigue was built (Figure 1). Since the ordinal SAS, AIS, and FS scores did not meet the multivariate normal distribution, the path coefficients in the model were estimated by a robust weighted least square method. In the SEM, all the measurement variables were involved as manifest variables. As described in Table 4, all the standardized path coefficients were statistically significant (*Ps* < 0.001). The effect value of PSU on sleep disturbance was 0.370. The total effect of PSU on physical fatigue was 0.385 including a direct effect of 0.177, and the indirect effect mediated by sleep disturbance was 0.208, which accounted for 54.03% of the total effect. The total effect of PSU on mental fatigue was 0.372 including a direct effect of 0.203, and the indirect effect mediated by sleep disturbance was 0.169, which accounted for 45.43% of the total effect. The bias-corrected bootstrap methods showed that all the 95% confidence intervals of the effect values did not include 0 indicating statistical significance. To test the stability of the mediating mode, we also involved the measurement variables as dichotomous scores according corresponding cutoffs and re-calculated the pathway coefficients. The parameter estimation results are summarized in Supplementary Table 1, and effect values were proved to be statistically significant.

**Table 4:** Path coefficients and effect values of PSU on sleep disturbance, physical fatigue, and mental fatigue.

**Pathway**	**β**	**SE**	***P-*value**	**Total effect (95% CI)**	**Direct effect (95% CI)**	**Indirect effect (95% CI)**
PSU → sleep disturbance	0.37	0.014	<0.001	0.370 (0.315, 0.420)	0.370 (0.315, 0.420)	–
PSU → physical fatigue	0.177	0.008	<0.001	0.385 (0.331, 0.435)	0.177 (0.127, 0.228)	0.208 (0.174, 0.238)
PSU → mental fatigue	0.203	0.006	<0.001	0.372 (0.322, 0.429)	0.203 (0.148, 0.259)	0.169 (0.143, 0.198)
Sleep disturbance → physical fatigue	0.562	0.018	<0.001	0.562 (0.514, 0.604)	0.562 (0.514, 0.604)	–
Sleep disturbance → mental fatigue	0.457	0.014	<0.001	0.457 (0.410, 0.505)	0.457 (0.410, 0.505)	–

*β: standardized regression coefficient*.

*PSU, problematic smartphone use; SE, standard error; CI: confidence interval*.

## Discussion

In the current study, we identified the relationship of PSU, sleep quality, and daytime fatigue among medical university students during the COVID-19 pandemic. We obtained an unoptimistic incidence of PSU (49.70%) among quarantined medical students, which was higher than that of Chinese senior students (16.4%) from an online survey conducted on February 2020 (8). Consistent with our results, a cross-sectional study revealed a PSU prevalence of 59.42% in medical students from two provinces in China (42), and in another study the prevalence was 43.3% among Chinese adults (43). According to the I-PACE model and CIUT, the high prevalence of PSU among quarantined medical students was related to various factors (44). Firstly, Beijing has initiated a top-level response to major public health emergencies at the time of investigation and adopted social isolation policies. University students mainly relied on the Internet to communicate with the outside world, including social networking, shopping, learning, etc., which might aggravate smartphone overuse. Secondly, medical students had heavy academic burden during the pandemic. They needed to complete various learning and research objectives through virtual education platforms. In addition, when in-person communication with tutors became less frequent, the pressure and anxiety level would also increase, which might be incentives of PSU. Researches have revealed that smartphone overuse was closely related to depression, anxiety, and other psychological disorders (12, 45), and these correlations have also been confirmed in several investigations conducted during the pandemic (11). Thirdly, with the continuous unfolding of the COVID-19 pandemic, college students are worried about the social stability and health status of their family and friends; thus, they have increased demand to browse online information through smartphones daily. A longitudinal ecological study from the United States showed that along with the unfold of COVID-19, college students used more smartphones, had less physical activity, and visited fewer outdoor places (20). In this study, we identified several types of college students who were more susceptible to have PSU during the pandemic, which was supported by the CIUT. Male, professional degree type, low income, living in rural, poor relationship with tutors and inactivity were potential risk factors. Among quarantined medical students, daytime fatigue was commonly observed (40.06%), of which the prevalence of physical and mental fatigue was 55.91 and 46.60%, respectively. In another cross-sectional study, the prevalence of fatigue was 67.3% among Chinese nursing students in post-COVID-19 era (46). We also found a high prevalence of sleep disturbance (70.57%) in this sample. A recent review study including 12,682 respondents showed that the pooled prevalence of insomnia was 30% in Chinese frontline healthcare workers during the COVID-19 pandemic (47). Several studies also reported various physical and mental health outcomes were associated with the pandemic among healthcare workers, adolescents, and adults (48–50). Similarly to this study, a recent web-based survey showed that Indian undergraduate and postgraduate medical students had high level of perceived stress and anxiety during the COVID-19 pandemic (51).

SAS-SV score was significantly correlated with AIS score (*r* = 0.38) and medical students with PSU were more likely to report sleep disturbance (OR = 2.91). Lateef and colleagues also found a statistically significant positive correlation between Internet addiction and insomnia among clinical medical students in Africa (52). We confirmed the *H-01* and the role of melatonin could account for the relationship between smartphone overuse and sleep deprivation, which has been confirmed by animal and human studies (29). Melatonin is one of the hormones secreted by the pineal gland, which helps sleep and regulates the circadian clock (53). The secretion of melatonin has an obvious circadian rhythm. Light stimulation during smartphone using at night will inhibit the activity of melatonin synthesis enzymes in the pineal gland, thus inhibiting the secretion of melatonin. We obtained significant correlations between SAS-SV score and FS score (*r* = 0.41), and *H-02* was supported. Subjects with PSU were more likely to experience physical fatigue (OR = 3.18) and mental fatigue (OR = 2.42). Compared with the previous review studies, we have obtained larger odds ratios in this study (16, 54), suggesting that the pandemic might exaggerate the excessive use of smartphones and the corresponding daytime fatigue and sleep disturbance. Overuse of smartphones can lead to worsen upper limb pain (14). People who have smartphone addiction are more susceptible to blurred vision, back pain, wrist pain, stiff neck, and other health issues (14, 55). A recent systemic review reported that excessive and frequent smartphone usage increases the risk of headaches by 38% (56). Previous studies have also confirmed that PSU is associated with fatigue and physical dysfunction in student and adult samples (37, 57). In addition, factors such as introversion personality and negative emotions highlighted by the I-PACE model also contribute to the association between Internet addiction behaviors and daytime dysfunction. We found sleep quality was significantly associated with both physical fatigue and mental fatigue, and *H-03* was supported. Firstly, although fatigue and sleep disturbance are defined as two independent, non-motor symptoms, they often overlap in clinical settings (58). People with fatigue often have difficulty in falling asleep and experience daytime sleepiness. In addition, the AIS and Pittsburgh Sleep Quality Index (PSQI), two most widely used sleep assessment scales, both contain components of daytime dysfunction, thus *H-03* was conceptually supported. Secondly, the AIS score was significantly correlated with physical fatigue (*r* = 0.58). Skeletal muscle is not only one of the most important motor organs, but also the peripheral clock organ closely related to circadian rhythm. More than 2300 genes in skeletal muscle are expressed with circadian rhythm (31). Laboratory studies have shown that when circadian rhythm is disrupted, skeletal muscle fiber type displacement, sarcomere structure changes, and mitochondrial dysfunction are observed (59). In particular, reduced mitochondrial biosynthesis ability is a key regulatory process leading to skeletal muscle dysfunction and reduced human endurance (60). Thirdly, abnormal cortisol and melatonin function might be the main physiological mechanism of the correlation between AIS score and mental fatigue (*r* = 0.48). Cortisol is a neuroendocrine hormone regulated by the hypothalamic-pituitary-adrenal cortex (HPA) axis, which can participate in body metabolism, activate the vitality of the nervous system, and regulate the function of the cardiovascular system (32). Circadian rhythm disorders can disrupt the secretion of cortisol, thereby weakening the body's ability to regulate the nervous system, leading to mental fatigue symptoms such as decreased daytime excitability, neurasthenia, and memory loss. Furthermore, *H-04* was confirmed in our SEM analysis. In the relationship of PSU and daytime fatigue, sleep quality mediated 50.03 and 45.43% of the total effect on physical and mental symptoms, respectively. The significance of path coefficients in the SEM model demonstrated the vital mediating role of sleep quality. The indirect effect of sleep quality in our hypothetical model could be supported by the above physiological mechanisms such as circadian rhythm and neurohormone secretion. Previous studies have also shown that sleep quality plays an intermediary role in PSU and physical and psychological illness such as eye symptoms, body dysfunction, and emotional problems in student samples (33, 61). Our findings have explored potential mechanisms of PSU on daytime function and are beneficial for health intervention among college students.

The emergence of new SARS-CoV-2 variants has dramatically increased the potential risk of future pandemics (62). Therefore, the government and colleges should alert to the adverse effects of excessive smartphone use on clinical health symptoms during the pandemic, and accessible psychological counseling services are necessary for quarantined university students. Tutors should strengthen the interaction with students, establishing timely and effective guidance on students' academic progress. While improving the virtual learning platforms, it is also feasible to monitor students' smartphone usage frequency during the lockdown. Establishing a monitoring network system that can send out reminders of smartphone overuse would benefit students' mental and physical wellness. Several limitations should be acknowledged. Firstly, the results did not indicate any causal inferences due to the cross-sectional design, and longitudinal studies are needed to further explore the COVID-19 pandemics' influence. Secondly, although the web-based survey and convenient sampling mode was the best option to reach subjects during the lockdown period, it inevitably led to selection bias and response bias. Thirdly, although we used standard measurements to identify the problematic use of smartphones, our data were not sufficient to discuss the duration and specific purpose of smartphones usage, which might overestimate the true incidence of PSU. Health-related information was collected from self-reports, which were less reliable than clinical diagnoses. Fourthly, the subjects of this study were exclusively from one city, and the extrapolation of the results needs to be treated with caution. However, Beijing can be regarded as a representative city of outbreak prevention and control in China, which to some extent reflects the trend of the whole country.

## Conclusions

In conclusion, during the pandemic of COVID-19, medical students in Beijing had serious smartphone overuse problems, which were associated with sleep disturbance, physical fatigue, and mental fatigue. Our study provided insights into the mechanism that sleep quality mediated the relationship between PSU and daytime fatigue, which was valuable evidence to suggest actions for maintaining university students' health status and constructing online education structures.

## Data Availability Statement

The original contributions presented in the study are included in the article/Supplementary Material, further inquiries can be directed to the corresponding author/s.

## Ethics Statement

The studies involving human participants were reviewed and approved by The Ethics Committee of Beijing Hospital. The patients/participants provided their written informed consent to participate in this study.

## Author Contributions

CZ, PZ, DL, and JJ proposed the concept and design. CZ analyzed and interpreted the data and wrote the manuscript. JT, SS, MZ, JC, and GZ drafted and edited the manuscript. CZ, DL, and JJ supervised the study and obtained funding. All authors read and approved the final version of the manuscript.

## Funding

This study was supported by the National Key R&D Program of China (Grant No. 2020YFC2002700), the Education and Teaching Research Project of Peking University Health Science Center (Grant No. 2020YB42), the Fundamental Research Funds for the Central University (Grant No. 3332021077), and Research Subject of Chinese Society for Academic Degree and Graduate Education (Grant No. A1-YX20180201-02).

## Conflict of Interest

The authors declare that the research was conducted in the absence of any commercial or financial relationships that could be construed as a potential conflict of interest.

## Publisher's Note

All claims expressed in this article are solely those of the authors and do not necessarily represent those of their affiliated organizations, or those of the publisher, the editors and the reviewers. Any product that may be evaluated in this article, or claim that may be made by its manufacturer, is not guaranteed or endorsed by the publisher.
