# Supplementary material for: Relationship of Problematic Smartphone Use, Sleep Quality, and Daytime Fatigue Among Quarantined Medical Students During the COVID-19 Pandemic
Source: Front Psychiatry. 2021 Nov 10;12:755059. doi: 10.3389/fpsyt.2021.755059 (PMC8631394; doi:10.3389/fpsyt.2021.755059)
Supplement: Supplementary file 1 [file Data_Sheet_1.docx]

**Supplementary Table 1．Path coefficients and effect values of PSU on sleep disturbance, physical fatigue, and mental fatigue**

| Pathway | β | SE | *P-*value | Total effect (95% CI) | Direct effect (95% CI) | Indirect effect (95% CI) |
| --- | --- | --- | --- | --- | --- | --- |
| PSU→ sleep disturbance | 0.370 | 0.014 | <0.001 | 0.370 (0.315, 0.420) | 0.370 (0.315, 0.420) | —— |
| PSU→ physical fatigue | 0.177 | 0.008 | <0.001 | 0.385 (0.331, 0.435) | 0.177 (0.127, 0.228) | 0.208 (0.174, 0.238) |
| PSU→ mental fatigue | 0.203 | 0.006 | <0.001 | 0.372 (0.322, 0.429) | 0.203 (0.148, 0.259) | 0.169 (0.143, 0.198) |
| Sleep disturbance→ physical fatigue | 0.562 | 0.018 | <0.001 | 0.562 (0.514, 0.604) | 0.562 (0.514, 0.604) | —— |
| Sleep disturbance→ mental fatigue | 0.457 | 0.014 | <0.001 | 0.457 (0.410, 0.505) | 0.457 (0.410, 0.505) | —— |

β: standardized regression coefficient.

Abbreviations: PSU, problematic smartphone use; SE, standard error; CI: confidence interval.

Note: Measurement scores were involved as dichotomous variables


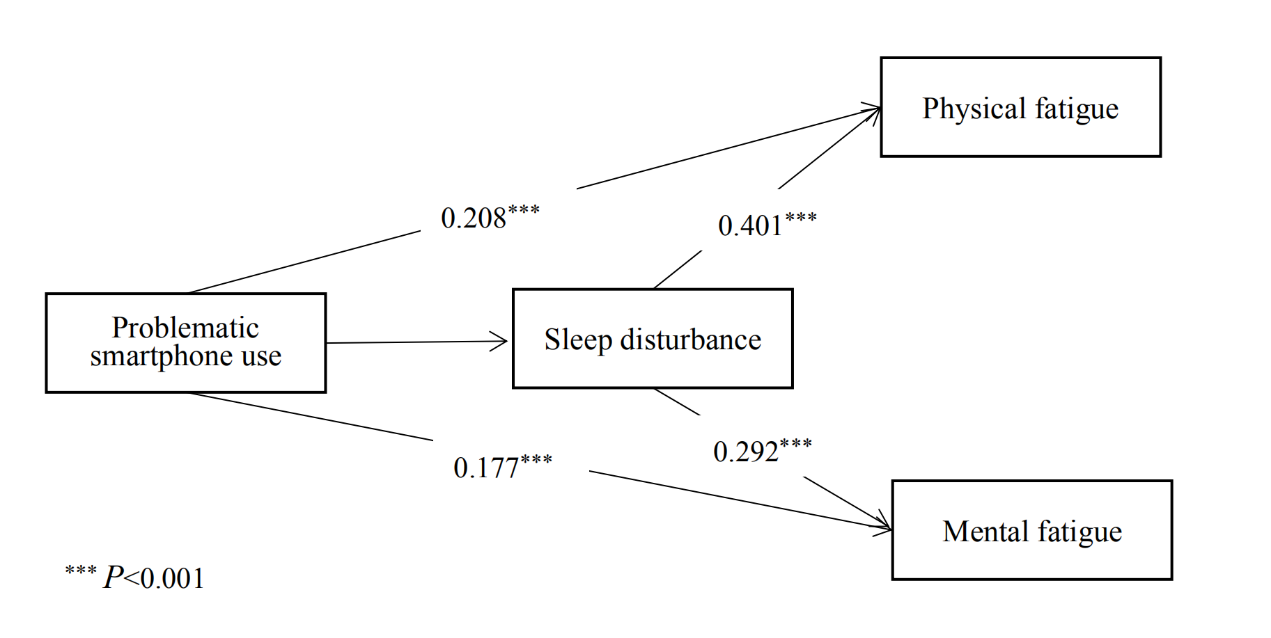


**Supplementary figure 1. Mediation models of problematic smartphone use, sleep disturbance, physical fatigue, and mental fatigue**
